# An Antibiotic-Releasing Bone Void Filling (ABVF) Putty for the Treatment of Osteomyelitis

**DOI:** 10.3390/ma13225080

**Published:** 2020-11-11

**Authors:** Raquib Hasan, Abbey Wohlers, Jacob Shreffler, Pranothi Mulinti, Hunter Ostlie, Codi Schaper, Benjamin Brooks, Amanda Brooks

**Affiliations:** 1 Department of Pharmaceutical Sciences, North Dakota State University, Fargo, ND 58102, USA; raquib.hasan@ndsu.edu (R.H.); jacob.shreffler@ndsu.edu (J.S.); pranothi.mulinti@ndsu.edu (P.M.); 2 Department of Pharmacy, North Dakota State University, Fargo, ND 58102, USA; abbey.wohlers@ndsu.edu; 3 School of Medicine, St. George’s University, University Centre Grenada, West Indies, Grenada; hostlie@sgu.edu; 4 College of Veterinary Medicine, Kansas State University, Manhattan, KS 66502, USA; schaper@vet.k-state.edu; 5 Department of Biomedical Sciences, Rocky Vista University, Ivins, UT 84734, USA; bbrooks@rvu.edu; 6 Department of Research and Scholarly Activity, Rocky Vista University, Ivins, UT 84734, USA

**Keywords:** osteomyelitis, vancomycin, vancomycin free-base, bone void filler, controlled drug delivery, bone regeneration, total joint replacement, bone biomaterials, infection, *Staphylococcus aureus*

## Abstract

The number of total joint replacements (TJR) is on the rise with a corresponding increase in the number of infected TJR, which necessitates revision surgeries. Current treatments with either non-biodegradable, antibiotic-releasing polymethylmethacrylate (PMMA) based bone cement, or systemic antibiotic after surgical debridement do not provide effective treatment due to fluctuating antibiotic levels at the site of infection. Here, we report a biodegradable, easy-to-use “press-fitting” antibiotic-releasing bone void filling (ABVF) putty that not only provides efficient antibiotic release kinetics at the site of infection but also allows efficient osseointegration. The ABVF formulation was prepared using poly (d,l-lactide-co-glycolide) (PLGA), polyethylene glycol (PEG), and polycaprolactone (PCL) as the polymer matrix, antibiotic vancomycin, and osseointegrating synthetic bone PRO OSTEON for bone-growth support. ABVF was homogenous, had a porous structure, was moldable, and showed putty-like mechanical properties. The ABVF putty released vancomycin for 6 weeks at therapeutic level. Furthermore, the released vancomycin showed in vitro antibacterial activity against *Staphylococcus aureus* for 6 weeks. Vancomycin was not toxic to osteoblasts. Finally, ABVF was biodegradable in vivo and showed an effective infection control with the treatment group showing significantly higher bone growth (*p* < 0.001) compared to the control group. The potential of infection treatment and osseointegration makes the ABVF putty a promising treatment option for osteomyelitis after TJR.

## 1. Introduction

Currently, in the United States over a million people undergo total joint replacements (TJR) each year [1,2,3]. This number is projected to exceed 4 million primary procedures each year by 2030 [2]. Although TJRs, including total knee replacements (TKR) and total hip replacements (THR), are arguably some of the most successful surgical practices to improve the quality of life, secondary or revision surgeries are becoming commonplace. Furthermore, with the success of TJRs, primary TJR procedures are occurring at an earlier age [4,5], often necessitating a revision during the patient’s lifetime as the prosthetic TJR materials age. According to the American Academy of Orthopedic Surgeons (AAOS), there is an increase in revision surgery. In fact, 365,000 secondary or revision procedures are projected by 2030 [2]. Aseptic loosening, implant age, and infection are major contributors to the rate of revision. Among the list of contributing factors, infection is a major, and perhaps the most concerning cause for TJR revision, responsible for 18% of revision THR and 26.8% of revision TKR. Infection also causes the majority of early implant failures (within 3 months of primary surgery) (18.9% for THR and 44.8% for TKR) [6,7]. Regardless of the underlying cause of the revision, the complexity of a revision procedure makes it less successful than primary TJR surgery [8]. The risk of infection after revision surgery is alarmingly high at 21% after septic revision; whereas, in the case of aseptic revisions the risk sits at 5.4% (compared to 1–2% after primary TJR) [9]. Unfortunately, implant removal and hardware replacement drives the risk of infection to a staggering 20–30% [10,11].

Osteomyelitis, a deep and progressive bone infection, is a clinically difficult to treat condition [12]. *Staphylococcus aureus* is the most common causative pathogen of prosthetic joint infection (PJI) after TJR, responsible for ~65% of total cases, out of which 20% are methicillin resistant *S. aureus* (MRSA) [7,13]. Current clinical treatment strategies include thorough surgical debridement followed by 4–6 weeks of parenteral antibiotic therapy, as well as implanting antibiotic-loaded polymethylmethacrylate (PMMA) based bone cement for local antibiotic delivery in bone void space [14]. Surgical debridement almost always leaves residual bacteria necessitating continuous, local antibacterial therapy for a prolonged period of time [15]. Notably, local antibiotic therapy at the site of infection not only provides a high local drug concentration but also helps avoid systemic side effects [14]. Such localized antibiotic therapy is often provided by antibiotic-releasing PMMA. Unfortunately, antibiotic-loaded PMMA has several disadvantages such as: Low antibiotic release rate (less than 10–15% of total loaded antibiotic release) [16,17,18] and a limited choice of antibiotics to be incorporated due to heat sensitivity [19]. Due to the poor biocompatibility and lack of biodegradability of PMMA, an additional surgery may bae needed for removal of PMMA followed by replacing it with a biodegradable bone graft [20]. In fact, inadequate drug release kinetics and sub-therapeutic dug concentrations eluted from PMMA may lead to antibiotic resistance [21], act as substratum for bacterial inoculation [22], and PMMA may act as a surface of biofilm growth [23]. Importantly, there is a current impetus to move towards cementless TJR compared to cemented TJR due to the longevity, mitigated failure, and higher bone density around the implant in the case of cementless TJR [24]. Developing an antibiotic-releasing bone void filler (ABVF) putty to fill defects created after debridement to prevent and cure infections after revision TJR [25] seems to be necessary to fulfill the role of an effective local antibiotic release system. Surgical debridement can leave voids in the bone that needs to be filled. A putty-like ABVF composition can meet this need due to the moldable nature and ease of use as a press fitting material to fill the void spaces. Moldability is considered as a critical property of bone graft substitutes [26,27]. Several of the bone graft substitute materials in the market have moldable putty-like property [26]. Moreover, biodegradable bone void fillers can work as a scaffold to guide tissue regeneration and infection regression as they degrade [28]. A putty-like ABVF that addresses the shortcomings of PMMA use and fulfills the abovementioned desired properties, seems to be a viable treatment option.

Vancomycin, formerly a “last line of defense” antibiotic, is now a common place in the treatment of osteomyelitis and its underlying pathogen, *S. aureus*, including MRSA [29,30]. Vancomycin is also well tolerated by human osteoblasts [31]. Unfortunately, when used parenterally, vancomycin has poor bone penetration and only a fraction of the drug reaches the bone having a bone/serum ratio of 10%, with even lower penetration in the osteomyelitic bone [32]. Furthermore, extended, high-dose intravenous therapy with vancomycin can lead to nephrotoxicity and Red man syndrome [33]. The local release of vancomycin not only can achieve high local antibiotic concentrations but also avoids systemic side effects. The current study evaluated the in vitro vancomycin release and in vivo potential of ABVF putty to be used to treat osteomyelitis, demonstrating antibacterial protection, moldability, biodegradability, as well as osseointegration and healing of local bone tissue.

## 2. Materials and Methods

### 2.1. Materials

PLGA (90:10) (poly (d,l-lactide-co-glycolide)) was purchased from Polysciences, Inc., Warington, PA, USA; PEG (5 kD) (polyethylene glycol) was purchased from Fluka, St. Louis, MO, USA; PCL (10 kD) (polycaprolactone) from Sigma-Aldrich, St. Louis, MO, USA; vancomycin hydrochloride (V-HCl) from Sagent Pharmaceuticals, Schaumburg, IL, USA; NMP (N-Methyl-2-pyrrolidone) from Fisher Sci, Pittsburg, PA, USA; CaCl_2_ (calcium chloride) from EMD Chemicals Inc., Gibbstown, NJ, USA; PRO OSTEON 500R was kindly provided by BIOMET, Parsippany, NJ, USA; MTT (3-(4,5-dimethylthiazol-2-yl)-2,5-diphenyltetrazolium bromide) from VWR, Radnor, PA, USA; TRIzol reagent from Life Tech, Carlsbad, CA, USA; and SuperScript™ VILO™ cDNA Synthesis Kit from Thermo-Fisher Scientific, Carlsbad, CA, USA.

### 2.2. Preparation of Vancomycin Free-Base (V-FB)

V-FB was made from V-HCl according to the previously published method [34]. Briefly, V-HCl was dissolved in water at a concentration of 70 mg/mL. NaOH (3N) was added to increase the pH to 8.00 and precipitate V-FB. After incubation of 30 min, the precipitated V-FB was centrifuged at 3000 rpm for 10 min. After washing the V-FB in sequential 70% ethanol and methanol, the V-FB was suspended in water, frozen, and lyophilized. The validation of V-FB was done by HPLC (Figure S1). The bioactivity of V-FB and V-HCl was also compared via a zone of inhibition (ZOI) assay (Figure S2).

### 2.3. Fabrication and In Vitro Characterization

#### 2.3.1. Preparation of ABVF

PRO OSTEON 500R was crushed and sieved to get a particle size distribution of 175 to 425 μm. The PRO OSTEON particles (350 mg) were soaked in a 1 mL V-HCl solution (100 mg/mL) and was dried at 37 °C. The polymers, PEG (21.2 mg) and PCL (42.5 mg), were melted and mixed at 65 °C in a steel slide mold. The previously V-HCL soaked and dried PRO OSTEON particles were added into the molten polymers to create a homogenous mixture. At this point, V-FB (vancomycin free-base) (55.5 mg) was added to the mixture. Subsequently, PLGA (85.5 mg) dissolved in 200 μL of N-Methyl-2-Pyrrolidone (NMP) and 22 mg CaCl_2_ (used as porogen) were added to the mixture. Phosphate buffered saline (PBS) was added (20 μL) dropwise to produce a putty-like consistency. Table 1 shows the % amount of each ingredient in the formulation. The prepared master mix was put into a 3D printed mold to get the desired size and cylindrical shape (4 mm diameter × 3.5 mm height) to be used for further studies.

#### 2.3.2. Scanning Electron Microscopy (SEM) of the ABVF Putty

SEM was done on the putty to observe the morphological characteristics of the outer and inner surface of ABVF. SEM images of the surface and the inside (exposed by cutting the ABVF with a razor blade) of the ABVF putty were taken after fabrication, one week of incubation in PBS and four weeks of incubation in PBS to observe the degradation of the putty. The samples were attached to cylindrical aluminum mounts with a colloidal silver paint (Structure Probe Inc., West Chester, PA, USA) followed by gold coating (Cressington Inc., Redding, CA, USA). Images were taken with a JEOL JSM-6490LV scanning electron microscope.

#### 2.3.3. Micro-Computed Tomography (μ-CT) of ABVF Putty

The sample was hot glued to a glass rod and placed into a GE Phoenix v|tome| xs X-ray computed tomography system with a 180 kV high power nanofocus X-ray tube xs|180 nf, high contrast GE DXR250RT flat panel detector, and molybdenum target (GE Sensing and Inspection Technologies GmbH, Wunstorf, Germany). One thousand projections were acquired at a voltage of 80 kV and a current of 300 µA. The voxel size was 6.4 µm. Acquired images were reconstructed into a volume data set using the GE datos|x 3D computer tomography software version 2.2 (GE Sensing and Inspection Technologies GmbH, Wunstorf, Germany). The reconstructed volume was then viewed and manipulated using VGStudio Max (Volume Graphics Inc., Charlotte, NC, USA).

#### 2.3.4. Mechanical Characterization

Mechanical compliance was characterized using a rheometer (ARG2, TA Instruments, New Castle, DE, USA). The ABVF putty was used to cover the bottom plate of the rheometer (25 mm diameter). Frequency was increased from 6.283 and 628.3 rad/s, and the strain within the viscoelastic region (determined by a strain sweep) was applied. In addition, a demonstration of its putty-like characteristic of ABVF was also performed by press-fitting the material into different 3D printed shapes.

#### 2.3.5. In Vitro Drug Release Kinetics

Drug release was determined by incubating the ABVF putty in PBS (phosphate buffered saline) release media. ABVF (4 mm × 3.5 mm) was put into 2 mL of PBS (1x) and was incubated at 37 °C. At different time points (day 1, day 2, day 3, day 7, and every week after that through 6 weeks), all of the release media was collected and replaced with fresh PBS. The vancomycin concentration in the release was measured by UV-Vis spectrometer (Spectramax m5, Molecular Devices, Downingtown, PA, USA) at 280 nm.

#### 2.3.6. In Vitro Antibacterial Activity

The in vitro antibacterial activity of the released drug was assessed against *Staphylococcus aureus* (ATCC 49230) using a Kirby-Bauer ZOI (zone of inhibition) assay [35]. Briefly, 100 μL of the collected drug release media was dried on a 6.5 mm diameter filter paper disk. An overnight *S. aureus* culture was prepared and diluted in PBS to get a 10^7^ CFU/mL bacterial concentration. The bacteria solution was streaked on TSA (trypticase soy agar) plates and the drug impregnated filter paper disks were placed on the plates (within 15 min of streaking) and incubated for 20 h at 37 °C. The ZOI around the disk was measured using a digital caliper.

#### 2.3.7. In Vitro Cytocompatibility of ABVF Putty

The in vitro cytocompatibility of ABVF putty was assessed via the MTT assay. Briefly, 100,000 MG-63 osteoblast cells (ATCC, Manassas, VA, USA) were seeded in each well of a 24-well plate. Cells were grown in Dulbecco’s Modified Eagle Media (DMEM) containing 10% fetal bovine serum (FBS) and 1% penicillin-streptomycin-fungizone (Lonza, Walkersville, MD, USA). Cells were incubated at 37 °C and 5% CO_2_. After reaching 60% confluence, 500 μL of collected release media from the ABVF drug release study in Section 2.3.5, was added to each well; release media from day 1, 7, and 21 was used. Control wells received PBS. After 48 h of incubation with the ABVF release media, cells were washed with PBS three times and the MTT reagent (0.5 mg/mL in PBS) was added. Subsequently, wells were incubated at 37 °C for 4 h. After incubation, the cells were washed with PBS and DMSO was added followed by 20 min of incubation in the dark. After this final incubation, the absorbance was read at 570 nm (Spectramax m5, Molecular Devices, Downingtown, PA, USA). Alternatively, to determine the direct effect of drug release on cells, ABVF was placed in transwell inserts in wells of 24 well plates and enough cell culture media was added to submerge the ABVF in the inserts. The control group contained the bone void filler putty containing no antibiotic in transwell inserts. After 48 h of incubation, the MTT assay was done to determine the cell viability as the described method above.

### 2.4. In Vivo Assessment

#### 2.4.1. Rat Osteomyelitis Model

In vivo efficacy of ABVF putty was evaluated in a rat osteomyelitis model under the supervision of the Institutional Animal Care and Use Committee at North Dakota State University (A19019). Male Sprague-Dawley rats (>350 g) were used for the in vivo study. Briefly, rats were anesthetized with isoflurane inhalation. Buprenorphine hydrochloride was injected subcutaneously as an analgesic. The right hind leg was shaved and sterilized using alcohol and iodine pads, repeatedly. A small incision of 12 mm was made below the knee over the tibial metaphysis. A 4.2 mm hole was drilled until it penetrated the marrow space of the tibial metaphysis using an orthopedic drill (Stryker, Kalamazoo, MI, USA). *S. aureus* (10^8^ CFU) in sterile saline was inoculated through the drill hole defect into the marrow space using a 25 μL Hamilton syringe followed by implantation of the ABVF putty (dimensions as descried in Section 2.3.1). The incision was closed using a series of mattress sutures followed by the application of surgical glue (Vetbond Tissue Adhesive, 3M, Saint Paul, MN, USA). The control group underwent the same surgical procedure but only received the putty without antibiotic. Rats were monitored daily for signs of discomfort and infection. After 10 weeks, the rats were euthanized, and the tibia was harvested for further study. Blood and kidney were also collected for further study.

#### 2.4.2. Serum Creatinine

Blood collected from the rats at the time of euthanasia was centrifuged at 2000#xD7; *g* (Allegra X-14R, Beckman Coulter, Brea, CA, USA) to collect the serum. The serum was used to measure the creatinine level using an enzymatic rat creatinine assay kit (Crystal Chem, Elk Grove Village, IL, USA) following the manufacturer’s protocol.

#### 2.4.3. X-ray and μ-CT

Radiographic analyses of the bone were done after disarticulating the limb and harvesting the bone following euthanasia. X-ray was done using the IDEXX CR Digital Radiography system (Westbrook, ME, USA) following standard protocols. Briefly, lateral and cranial-caudal radiographic images were obtained of each limb at mAs: 4 and kVp: 40. The μ-CT of the bone was done following a similar procedure as described in Section 2.3.3 with a slight adjustment of the current to 400–500 µA.

#### 2.4.4. Bone Volume

Bone volume was determined based on the μ-CT scanned bones using the VGStudio Max (Volume Graphics Inc., Charlotte, NC, USA) software. A region of interest (ROI) was set on each of the scanned bones. Adjusting the contrast to highlight the bone in the ROI and comparing the ROI from the infection control group and the treatment group determined the amount of regenerated or new bone growth.

#### 2.4.5. Histology

Rats were euthanized after 10 weeks. The harvested bones were fixed in 10% neutral buffered formalin for 72 h. The bones were decalcified by immersing in an EDTA solution for 2 weeks, with a solution exchange every other day. Taking an X-ray of the bone ensured the decalcification end-point. The bone was sectioned after embedding it in paraffin wax. The sections were mounted on glass slides and stained with either an H&E stain or gram stain according to standard protocols. Briefly, the sections were deparaffinized in Clear Rite 3 (Thermo Fisher Scientific, Kalamazoo, MI, USA). Subsequently, the tissue was rehydrated with a decreasing gradient of ethanol. After H&E staining (Scy Tek Lab., Logan, UT, USA), the tissue section was covered with a glass coverslip using synthetic resin. Alternatively, gram staining was done according to the manufacturer’s protocol for the BD gram staining kit (Sparks, MD, USA) with slight modification. Briefly, the slide was dipped in gram crystal violet for 20 s followed by washing in cold tap water. Subsequently, slides were dipped into stabilized gram iodine for 20 s followed by washing in tap water. Gram decolorizer was used until the slides were colorless followed by washing in cold tap water. Slides were dipped in gram safranin for 15 s followed by washing in cold tap water. Slides were bolted to dry, and a cover slip was added.

#### 2.4.6. Bacterial Colony Count

The collected bone was flash frozen in liquid nitrogen, followed by pulverization using a custom-made bone crusher (Figure S3). The pulverized bone was weighed and suspended in 500 μL of PBS and vortexed for 1 min. Serial dilutions were made [34] and 10 μL of suspension was plated on blood agar plates (Fisher Sci, Pittsburg, PA, USA). Subsequently, plates were incubated for 48 h at 37 °C and bacterial colonies were counted. To get the total number of bacteria in the total sample, the number of colonies were multiplied by the dilution factor and normalized per gram of bone.

#### 2.4.7. Polymerase Chain Reaction (PCR)

PCR was done to identify the presence of inoculated bacteria. Bacterial RNA was isolated from the pulverized bone samples using TRIZOL according to the manufacturer’s instructions for RNA isolation [36,37]. The extracted RNA was used to synthesize first strand cDNA using the SuperScript™ VILO™ cDNA Synthesis Kit according to the manufacturer’s protocol. Primers (forward primer: GCACATCTTGACGGTACCTAAT and reverse primer: CGGGACTTAACCCAACATCTC) were designed to amplify 16 s RNA specific for *S. aureus* (ATCC 49230) (PCR program: 95 °C (2 min) for initial denaturation, 40 cycles of 95 °C (30 s), 54 °C (30 s), 72 °C (30 s), and final extension at 72 °C (5 min)). Primers were synthesized by Integrated DNA Technologies, Inc., Coralville, IA, USA. PCR was run using the Go-Taq green master mix (Promega, Madison, WI, USA) on a MultiGene™ OptiMax thermal cycler (Labnet Inc., Edison, NJ, USA). For each PCR reaction, 240 ng of cDNA was used. PCR products were examined by electrophoresis in a 1% (*w*/*v*) agarose gel and visualized by ethidium bromide.

### 2.5. Statistical Analysis

GraphPad Prism (version 8.4.3) was used for graphs, calculations, and statistical analyses. Student’s *t*-test using an α = 0.05 was done to determine the statistical significance for experiments containing *n* = 2 groups. One-way ANOVA was done to determine the statistical significance for experiments containing *n* > 2 groups using an α = 0.05.

## 3. Results

### 3.1. Scanning Electron Microscopy (SEM) of the ABVF Putty

**Figure 1:**
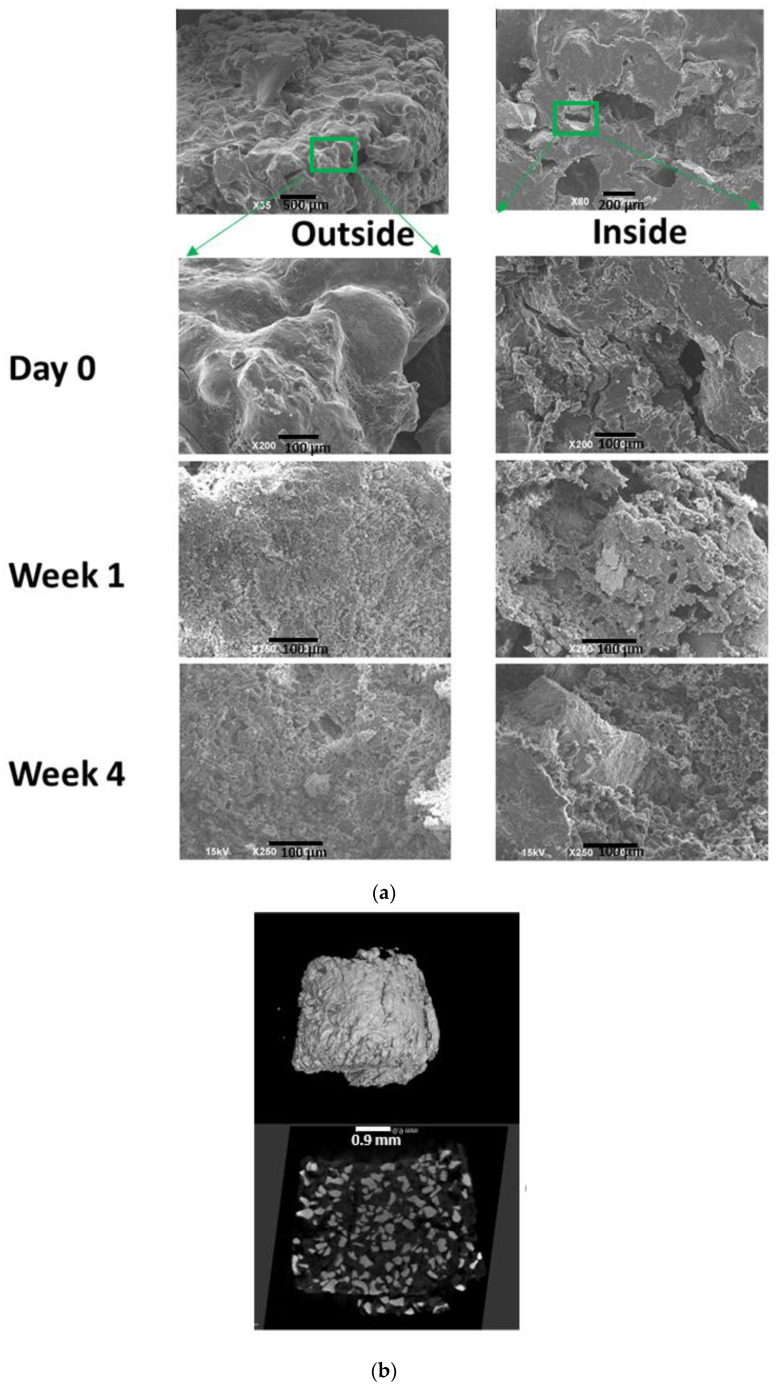
(**a**) SEM image of ABVF after manufacturing. The outer surface is rough (top left corner) and the inner surface has pores (top right corner). SEM images of ABVF degradation in phosphate buffered saline at different time points, showing increasing signs of degradation on both the outer and inner surfaces of the ABVF. (**b**) The micro-computed tomography image of ABVF shows a homogenous distribution of the PRO OSTEON particles in the polymer matrix.

SEM was done to see the morphology of both the outer and inner surfaces of the ABVF putty. SEM images show that ABVF has a rough outer surface (Figure 1a) while the inner surface has both macro and micro porous structures (Figure 1a). In vitro degradation of the ABVF putty over time was also monitored via SEM. SEM was done at day 0, and at day 7 and day 28 after incubation in PBS (Figure 1a). The SEM images show continuous degradation of ABVF after exposure to PBS. The outer surface showed signs of degradation at day 7. At day 28 there were signs of erosion and pore formation on the outer surface due to degradation. The inner surface also showed signs of degradation and pore formation.

### 3.2. The μ-CT of ABVF Putty

In vitro μ-CT was done to see the homogeneity of the formulation. Images show that the PRO OSTEON particles were homogenously mixed and distributed within the polymeric matrix (Figure 1b). The percentage weight of PRO OSTEON was 52% of the total weight of the ABVF putty. The images ensured that not only does the manufacturing of the ABVF putty produce a homogenous mixture of the ingredients but also that the process is reproducible as the ABVF putty from different batches showed similar PRO OSTEON particle distribution the polymer matrix.

### 3.3. Putty-Like Mechanical Property

**Figure 2:**
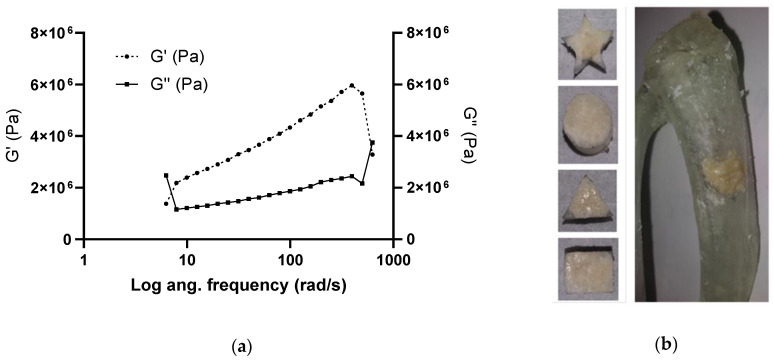
(**a**) Storage (G′) and loss modulus (G′′) of ABVF putty at an increasing angular frequency at room temperature (25 °C). (**b**) Press-fitting of ABVF putty in different 3D printed shapes and in a defect created in a 3D printed rat tibia. The ABVF putty could be easily press-fitted into shapes and seemed to contact the surrounding surfaces completely, without leaving any void spaces.

To evaluate the putty-like property of the ABVF, rheology was done (*n* = 3). From Figure 2a, it is clear that the loss modulus (G′′) increases as the angular frequency increases, which is characteristic of the putty-like material [38]. As frequency continued to increase, the storage modulus G′ also increased. This increasing G′ signifies an elastic-solid-like behavior [39]. The ABVF putty also could be easily molded into different 3D printed shapes (Figure 2b), demonstrating good press fitting, an important handling property for clinical use. During press fitting, the sticky ABVF stayed in place and maintained its structural integrity. Later, to simulate in vivo bone void space filling, a defect was made in a 3D printed rat tibia and ABVF putty could be press-fitted into the defect without leaving any visible void space.

### 3.4. In Vitro Vancomycin Release Kinetics

**Figure 3:**
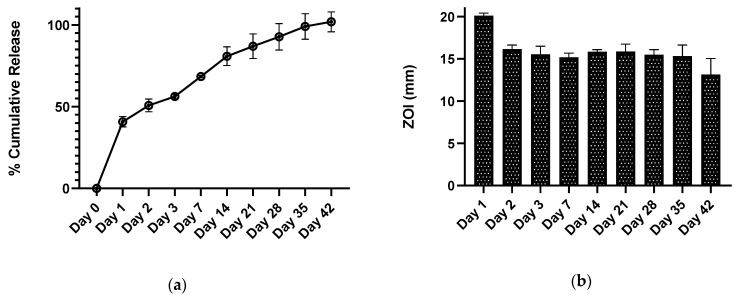
(**a**) Drug release kinetics of vancomycin from the ABVF putty shows drug release for up to 42 days (6 weeks). (**b**) The bioactivity of released vancomycin was determined via a Kirby Bauer zone of inhibition study against Staphylococcus aureus (strain ATCC 49230: 1.0 × 10^7^ CFU/mL).

An in vitro drug release study was done to assess the drug release over the desired 6-week period. The cumulative drug release curve of the ABVF formulation (Figure 3a), which best fits the Korsemeyer-Peppas model (Supplementary Table S1), showed the desired drug release kinetics over 6 weeks. It was observed that an initial burst release of 40% occurred during the first day of release. That may be due to the drug present near the surface of ABVF. Later, a more sustained release was observed, indicating that the vancomycin was released from the ABVF putty through both erosion of the polymer matrix and diffusion as the release media penetrates into the putty [35]. Erosion was also evident from the SEM images of degradation where erosion and degradation of the polymer matrix created pores (Figure 1a), which also might be conducive for diffusion.

### 3.5. In Vitro Antibacterial Activity

The in vitro antimicrobial activity was assessed via the ZOI assay against *S. aureus* (ATCC 49230). The released vancomycin showed antimicrobial activity during the entire study period of 6 weeks (Figure 3b). Notably, vancomycin-HCl and (V-FB) showed similar bioactivity at the same concentration in the ZOI assay (Figure S2).

### 3.6. In Vitro Cytocompatibility of ABVF Putty

**Figure 4:**
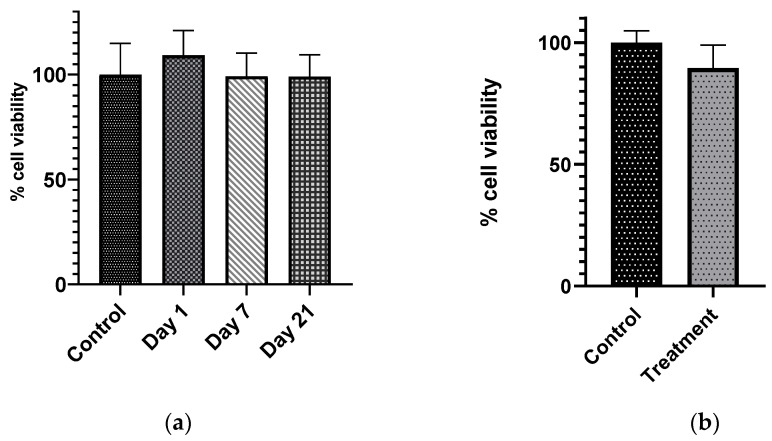
(**a**) There was no significant decrease in the cell viability of MG63 cells when the cells were exposed to different drug release samples from different time points, day = 1, 7, and 21 (α = 0.05, *n* = 4), compared to the control cell receiving only phosphate buffered saline. (**b**) When the drug was released from ABVF placed inside transwell inserts, the treatment cells did not show any significant difference compared to the control cell (α = 0.05, *n* = 3).

To assess whether the released vancomycin is tolerated by the osteoblast cells, cytocompatibility was evaluated via the MTT assay. The MTT assay showed that there was no significant difference in cell viability between the control and drug release group (Figure 4a). Furthermore, when the ABVF putty was placed in transwell on top of the MG63 osteoblasts culture to release drug directly on cells, the cell viability assay showed no significant difference in the cell viability between this treatment group cells and control cells – for which blank bone void filler was placed in transwell on top of the cells (Figure 4b). This indicaticated that the amount of vancomycin released was well tolerated by MG63 osteoblasts cells.

### 3.7. In Vivo Study Results

Following extensive in vitro characterization and validation, the ABVF putty was implanted into a rat orthopedic drill hole defect model. Thirteen rats were implanted with the ABVF putty while 12 rats were implanted with the BVF putty with no antibiotic. A spontaneous fracture was seen in three rats (within 1 week of the surgery) necessitating euthanasia and removal from the study cohort. All of these three rats were in the control group. On average, surgeries took about 15 min from incision to suturing the wound. In the case of the treatment group, swelling and/or redness at the surgical site seemed to resolve in about one week post-surgery. However, in the control group, some rats showed drainage from the wound. Nevertheless, the temperature of all the rats seemed to be normal, similar to healthy rats (~36° C), and all the rats were weight bearing and steadily gained weight during the experiment.

#### 3.7.1. Serum Creatinine

**Figure 5:**
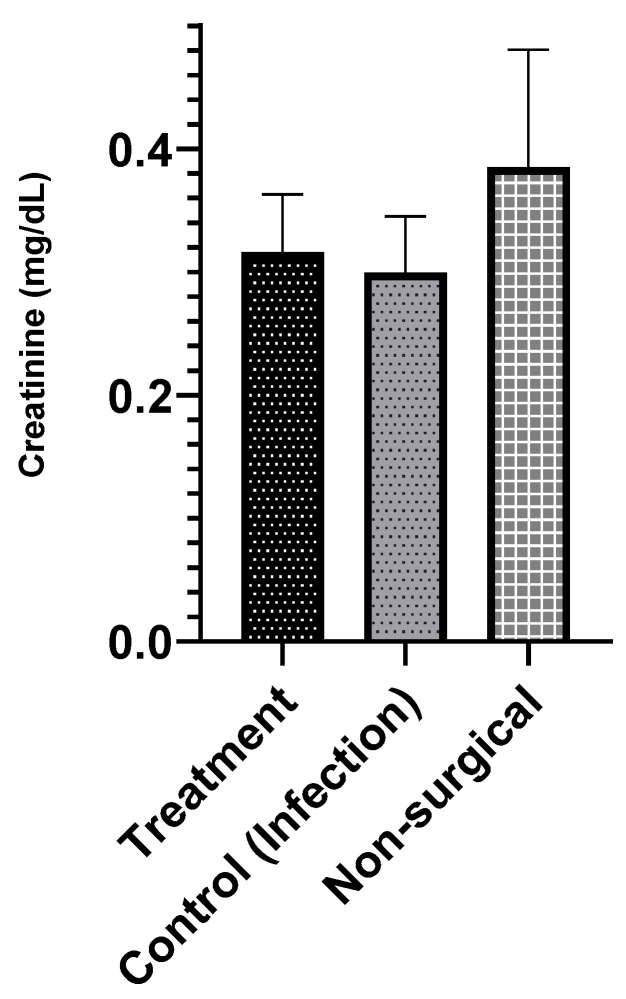
Serum creatinine level shows no significant difference between the infection treatment (*n* = 4), infection control (*n* = 6), and non-surgical (no infection) control groups (*n* = 3). The serum creatinine level seems to be within normal limits (α = 0.05).

Although there was no evidence of cytotoxicity in vitro, vancomycin can potentially cause nephrotoxicity [40], especially in the case of prolonged intravenous (IV) treatment, during elimination. Thus, serum creatinine levels were measured to assess if locally released vancomycin at the surgical site had any nephrotoxicity [41]. Serum creatinine levels in the infection treatment, infection control, and no surgery control groups were within the previously reported range of 0.3–0.6 mg/dL (Figure 5) [42,43], and there was no significant difference between the groups.

#### 3.7.2. Radiographs—X-ray and μ-CT

**Figure 6:**
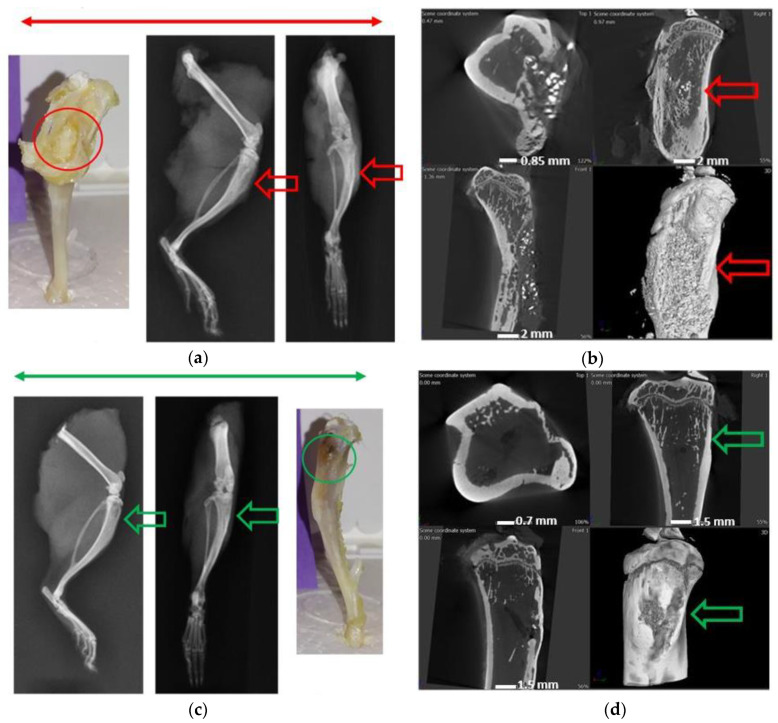
(**a**) Images under the red bar show the osteomyelitic bone in the infection control group. The macroscopic view (in red circle) and X-ray images (red arrow) show signs of osteomyelitis and sinus formation to drain the puss. (**b**) The μ-CT images show a deformed bone and narrowing of marrow space in this control group. (**c**) Images under the green bar show the treatment group animals with no apparent signs of osteomyelitis (green circle). (**d**) The μ-CT images show signs of bone healing and remodeling (green arrows).

Macroscopically the bones of the infection control group showed the presence of purulent discharge and sinus tracts formation. Bone deformity, periosteal reaction, narrowing of marrow space, and presence of fibrous tissue was also visible around the surgical sight (Figure 6a,b). All of the observations corresponded to the presence of osteomyelitis in the infection control group. In the treatment group (Figure 6c,d), signs of a healing callous, no visible implant, and ongoing bone remodeling were observed. Additionally, no fibrous tissue or the presence of inflammation or purulent discharge was observed. The μ-CT images showed signs of bone remodeling, and the marrow space was re-established.

#### 3.7.3. Bone Volume Measurement

**Figure 7:**
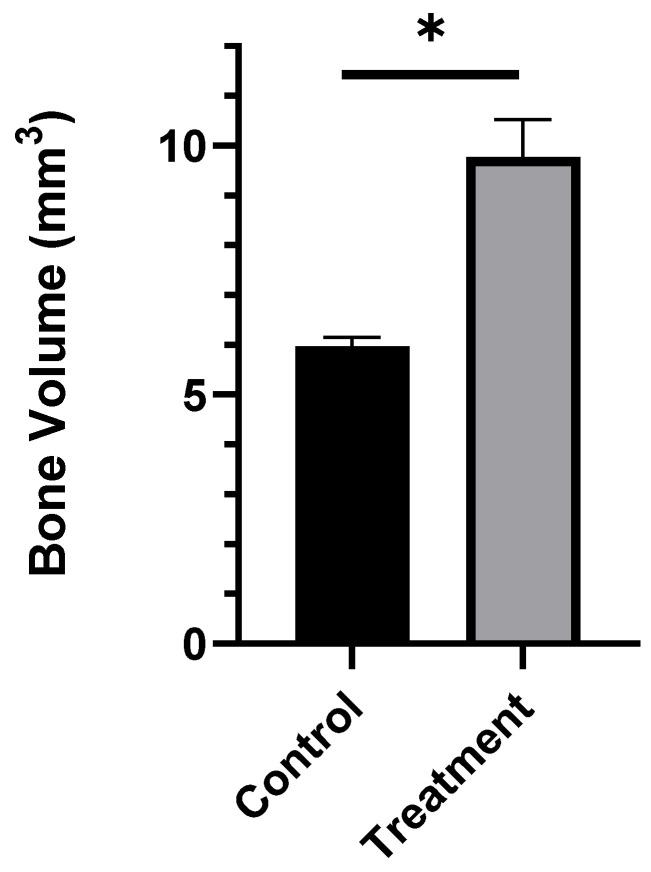
The bone volume in the treatment group (calculated from the µ-CT image) was significantly higher compared to the control group (*n* = 3). * *p* < 0.001.

The bone volume was measured after selecting the surgical site as the region of interest (ROI). In the ROI, the treatment group had a significantly higher amount of bone volume compared to the infection control group (*p* < 0.001, Figure 7). ABVF caused the eradication of infection signs and functioned as a scaffold for host bone integration and growth.

#### 3.7.4. Histology

**Figure 8:**
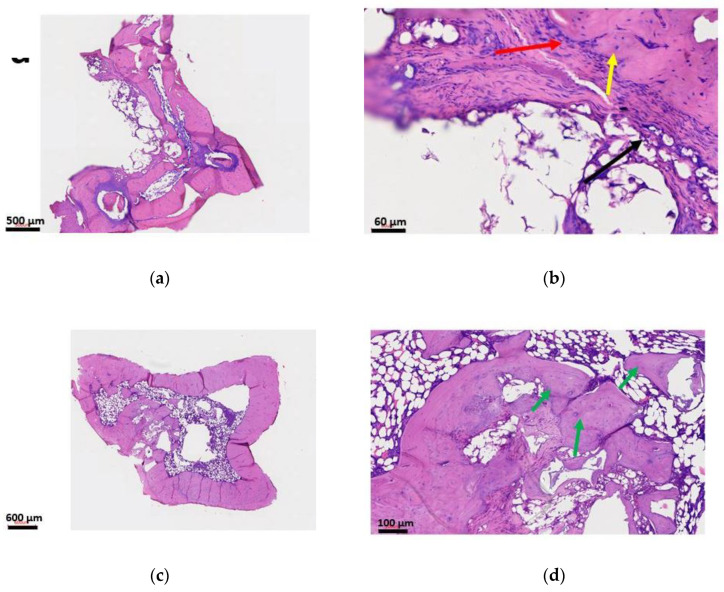
H&E staining of rat tibia. Images (**a**,**b**) are from the infection control group showing signs of osteomyelitis. Narrowing of the marrow space and destruction of the bone is visible. In image (**b**), the red arrow indicates an inflammatory response, whereas the yellow arrow shows an osteoclast and the black arrow shows fibrosing with granulation. In images (**c**,**d**), the treatment group shows no signs of osteomyelitis. The bone seems to be healing and the implanted ABVF was degraded. The new bone grew into the space that was occupied by ABVF. The green arrow in image (**d**) is showing new bone growth.

H&E staining shows signs of osteomyelitis in the infection control group (Figure 8a,b). Major bone destruction, narrowing of the marrow space, and fibrous tissue formation is abundant. In the treatment group (Figure 8c,d), no signs of osteomyelitis were visible. The ABVF putty seemed to have degraded, leaving space for host bone ingrowth and facilitating osteointegration and bone healing.

#### 3.7.5. Bacterial Load

**Figure 9:**
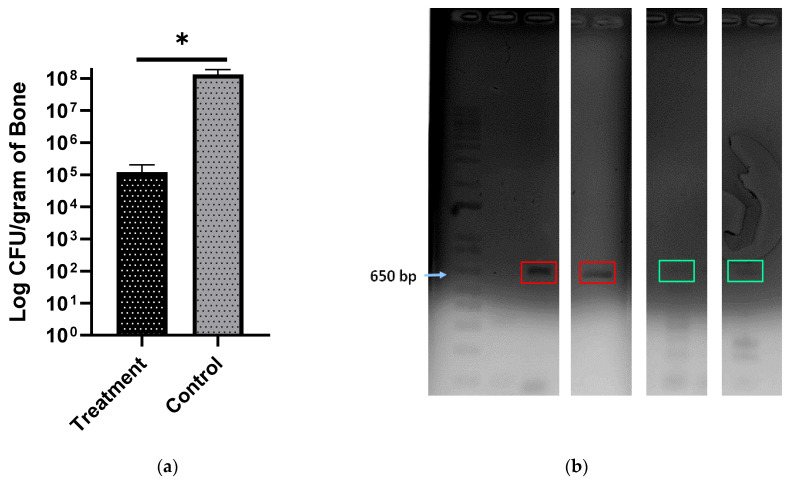
(**a**) Bacterial load per gram of bone in the control and treatment groups. At *p* < 0.0003, the bacterial load is significantly lower in the treatment group (*n* = 6) than in the control group (*n* = 4); * *p* < 0.0003. (**b**) DNA gel images of PCR amplified 16S from *S. aureus*. The red boxes show DNA bands from control group animals and the bands in the green box are from the treatment group.

Based on colony counts, the bacterial load per gram of bone was found to be significantly lower in the treatment group compared to the control group (Figure 9a). There was a 3-log reduction in the bacterial load in the treatment group, which is clinically significant. Histology and radiographs did not show any signs of infection in the treatment group animals even after the 10-week experimental time course, although a minimal number of bacterial colonies were still visible upon culture.

#### 3.7.6. PCR

Furthermore, PCR was done to confirm the strain of pathogenic bacteria in the bone and the inoculated species were the same. After extracting RNA and synthesizing cDNA from it, SA-1 primers specific for 16S rRNA of *S. aureus* (ATCC 49230) were used to amplify a 600 bp pair band and to confirm the presence of *S. aureus* (ATCC 49230). The band was clearly seen in the control culture as well as the control animals and only lightly seen in the test animals (Figure 9b), potentially an indication of decreased bacterial load, although it must be noted that this technique cannot assess the viability of the bacteria present.

## 4. Discussion

Osteomyelitis after TJR is a difficult clinical challenge due to poor pharmacokinetics in the bone; additionally, the presence of hardware can lead to stress shielding and bone necrosis and prevalent sequestra that provide a safe environment to harbor pathogenic bacteria, allowing almost unfettered proliferation [44]. PMMA based bone cements and beads are the standard of care for revision surgeries to treat osteomyelitis after TJR despite several drawbacks, including high temperature of processing, non-biodegradability, and poor drug release kinetics. The polymerization of the methymethacrylate monomers to create PMMA cement produces an exothermic reaction reaching a high temperature (115.2 °C) during the curing process, [45] not only limiting the candidate biomolecules that can be incorporated but also potentially causing the surrounding tissue necrosis. ABVF putty fabrication as described herein, is not an exothermic process, allowing an expanded repertoire of drugs to be potentially added. In the current study, two different forms of vancomycin were incorporated into the ABVF putty and sustained drug release was achieved (Figure 3a). Furthermore, inadequate antibiotic pharmacokinetics and antibiotic drug release kinetics can also lead to the development of resistant bacteria and exacerbate an already challenging clinical treatment. The literature has reported that antibiotic-releasing PMMA based systems have a high burst release with little sustained release. In fact, some reports indicate that only about 10% of the drug gets released [46] with the remaining drug leaching below the antibiotic’s minimum inhibitory concentration for a prolonged period, potentially leading to antibiotic resistance [47]. Finally, PMMA is not biodegradable and may act as a foreign body and nidus of subsequent infections. The ABVF putty reported here released almost all the drug payload within 6 weeks (Figure 3a) and biodegraded (Figure 1a). Several biodegradable and osseointegrating materials have been investigated to locally deliver antibiotics. Calcium sulfate is a popular one with variable results. Studies showed very quick in vitro exhaustion of vancomycin load from calcium sulfate beads within 3 days to more sustained in vitro release over 6 weeks; while no in vivo efficacy was reported [18,48]. Another study using calcium phosphate—calcium sulfate composite beads to deliver vancomycin showed release up to 22 days [49]. These studies used different sampling methods which may lead to variability [48]. Researchers investigating delivering gentamicin from hydrogel and bovine bone substitute based composites [50] or, chitosan and bovine bone substitute based composites [51] could not sustain gentamicin release for more than 1 day and 2 weeks, respectively, while not fully eliminating bacteria in in vitro models. Nonetheless, using these materials to deliver antibiotics can be very challenging due to the extremely high variability. ABVF controlled the antibiotic delivery from a polymer-substrate composite matrix to address the challenge of variable antibiotic release, and provided an antibacterial activity against *S. aureus* for 6 weeks (Figure 3b).

Previous studies with implantable/injectable biodegradable ceramic and/or, polymer-based vancomycin delivery systems showed varying results. Coelho et al. developed a controlled vancomycin releasing bone substitute using heparinized nanohydroxyapatite and collagen, which released an inhibitory concentration of vancomycin against *S. aureus* only for 9 days [31]. Later, Padrāo et al. showed another heparinized nanohydroxyapatite and collagen-based bone substitute to deliver quantifiable vancomycin for 19 days. Although the authors showed that the day 1 release of vancomycin was bactericidal, they did not show if the released vancomycin at all timepoints were able to eradicate the bacteria [52]. Le Ray et al. used vancomycin-loaded PCL particles to treat osteomyelitis in a rabbit model (10^7^ CFU *S. aureus* inoculation) with a 2-log reduction in bacterial count after 110 days of treatment [53], although no osseointegrating material was used in this study. In another study, Inzana et al. reported that when the vancomycin-loaded calcium phosphate was implanted, it resulted in around 1-log reduction in bacterial count in the bone [54]. In the current study, ABVF was able to provide at least a 3-log reduction in bacteria in the treatment group in vivo throughout the 10-week study course (Figure 9a). Notably, a minimal number of colonies were evident in some of our replicates, likely due to contamination from the normal skin flora during sample preparation. The skin swab of the euthanized rats showed the presence of bacteria in the culture, although the amount was not quantified (data not shown). However, it could not be confirmed if the source of bacteria in some of the bone extract culture plates were from the bone or from the contamination from the skin while harvesting. Nonetheless, the bacteria in the culture were identified via PCR as *S. aureus* 49230 (Figure 8b), the species inoculated into the defect. Other studies containing vancomycin HCl and vancomycin free-base in polyurethane scaffolds, also show remnant bacteria in the bone although the bacterial load used in those studies was much lower (10^5^ CFU *S. aureus*) than in our study (10^8^ CFU *S. aureus*) [34,55]. In the current study, even after 10 weeks in the treatment group, no signs of infection were observed in radiology and histology (Figure 6 and Figure 8). It can be argued that the absence of clinical signs of infection such as inflammation, bone destruction, pus formation, etc. can be considered as an effective treatment against the infection as the active infection would have compromised the bone regrowth [56], which we did not observe in our study. The residual bacterial presence seen, despite the lack of evidence of infection in rat bones, could also be due to cross-contamination from using a single bone crusher for pulverizing bones from different rats, with cleaning the bone crusher with only 70% alcohol. Using separate bone crushers could have addressed this shortcoming of the study.

The local delivery of vancomycin may address the challenge of some of the systemic, extended-use related toxicities of this antibiotic (e.g., nephrotoxicity, ototoxicity, and poor venous compatibility) [53]. The serum creatinine level was assessed in the in vivo study, which indicated that no nephrotoxic effects of locally released vancomycin from the ABVF putty (Figure 5). Importantly, the released vancomycin was non-cytotoxic on host cells (Figure 4 a,b).

The treatment of osteomyelitis aims not only to manage the infection but also to achieve bone regeneration [57]. These two goals must be matched as they are intimately connected by the “race for the surface” [58] with delayed integration being associated with increased infection [59]. Current bone void fillers frequently suffer from limitations in their ability to integrate with the host bone, either based on their biodegradation, particle size, or contact with the host bone. In our study, the biodegradable ABVF allowed new bone growth in the implanted space as well as showed signs of osseointegration (Figure 6 and Figure 8), which would ensure proper healing of the infected site.

Osseointegration can be limited by the particle size of the mineral composition in other hyaluronic acid (HA) and calcium phosphate (CP) based Bone Void Fillers (BVF) as the particle size in ceramic/polymer composite BVF plays an important role in bone healing. Previously, it was thought that the small bone graft particle of <105 µm would provide better healing due to the greater surface area. However, in a recent study, it was found that BVFs with particle sizes ranging between 100–500 µm showed 12–16% more new bone growth compared to BVF with smaller (<105 µm) particles in a rabbit femoral condyle defect model [60]; an observation corroborated by other studies. In another study, particles between 100–300 µm showed significantly more new bone formation and faster resorption compared to particles ranging from 1000–2000 µm in size when used in a femoral defect model in rhesus monkeys [61]. Finally, in a study on the effect of particle size on bone healing in a rabbit femoral condyle defect model, it was shown that animals receiving bone graft particles in the range of 250–500 µm experienced significantly more new bone growth when compared to groups receiving larger (1000–2000 µm) or smaller (90–125 µm) particles in a composite BVF [62]. The ABVF putty reported here contained the PRO OSTEON particles of 175–425 µm to maximize bone regrowth and host bone integration.

In rats treated with the ABVF putty composition, bone regeneration and ingrowth were significantly higher compared to the control group (*p* < 0.001) (Figure 7). This integration may have resulted from the intimate contact between the host bone and the ABVF putty composition. In recent years, injectable or settable BVFs have gained popularity [60] as they are easy to use and when applied can settle and harden at the site of application. Our ABVF composition demonstrated a desirable, putty-like behavior that allowed easy press-fitting (Figure 2), and which was also demonstrated by the increasing loss modulus (G′′) indicative of a viscous response and flow of the material [63]. Furthermore, the increasing loss modulus G′ signifies an elastic-solid-like behavior [39], which indicates stiffening of the ABVF over time. The increase in G′ was also higher than G” as the shear stress increased showing a shift towards stiffening under higher stress corresponding to the putty-like behavior [63]. The in situ hardening behavior of the ABVF putty is also desirable as it would remain in the defect site. Finally, hardening of ABVF putty overtime was also observed during the in vitro release in an aqueous environment. Theoretically, this phenomenon may be accounted for by PLGA dissolved in NMP, which shows phase inversion. In an aqueous environment, the NMP diffuses out of the polymer leaving a hardened matrix behind [64], eliminating the need for ex vivo curing prior to the wound closure.

## 5. Conclusions

The ABVF putty has shown a sustained vancomycin release demonstrating efficacious in vitro and in vivo antibacterial activity. The ABVF putty was biodegradable and supported bone growth. The ABVF putty developed here is a promising new alternative for treating osteomyelitis after revision TJR. Future work will be focused on the use of different osteoconductive materials in the ABVF putty to assess the effect on bone regrowth.

## Figures and Tables

**Table 1:** The % amount of ingredients in the antibiotic-releasing bone void filler (ABVF) formulation.

Ingredients	PRO OSTEON	V-HCl	V-FB	PLGA	PCL	PEG	CaCl_2_
**% amount**	51.72	14.78	8.20	12.63	6.28	3.13	3.25

## Supplementary Materials

The following are available online at https://www.mdpi.com/1996-1944/13/22/5080/s1, Figure S1.1: HPLC validation of V-fb. The peaks appeared at the same time for both V-HCl and V-fb confirming the production of V-fb, Figure S1.2: The bioactivity of V-HCl and V-FB was determined via a Kirby Bauer zone of inhibition study (Staphylococcus aureus strain 49230: 1.0 × 10^7^ CFU/mL), Figure S1.3: The bone crusher was made using three components. The bottom part (A) screws on to the barrel (B). The piston (C) then can be used to pulverize the bone using a hammer. (D) is the finished crusher, Table S1: Vancomycin release kinetics from ABVF fitted into different kinetics equation. Korsemeyer-Peppas equation seems to have the best fitted model with high R2 value.
