# Supplementary material for: An Antibiotic-Releasing Bone Void Filling (ABVF) Putty for the Treatment of Osteomyelitis
Source: Materials (Basel). 2020 Nov 11;13(22):5080. doi: 10.3390/ma13225080 (PMC7698155; doi:10.3390/ma13225080)
Supplement: Supplementary file 1 [file materials-13-05080-s001.pdf]

materials

# Supplementary Materials: An Antibiotic-Releasing Bone Void Filling (ABVF) Putty for the Treatment of Osteomyelitis

**Raquib Hasan [1], Abbey Wohlers [2], Jacob Shreffler [1], Pranothi Mulinti [1], Hunter Ostlie [3], Codi Schaper [4], Benjamin Brooks [5] and Amanda Brooks [1,6*]**

[1] Department of Pharmaceutical Sciences, North Dakota State University, Fargo, ND 58102, USA; raquib.hasan@ndsu.edu (R.H.); jacob.shreffler@ndsu.edu (J.S.); pranothi.mulinti@ndsu.edu (P.M.)
[2] Department of Pharmacy, North Dakota State University, Fargo, ND 58102, USA; abbey.wohlers@ndsu.edu
[3] School of Medicine, St. George's University, University Centre Grenada, West Indies, Grenada; hostlie@sgu.edu
[4] College of Veterinary Medicine, Kansas State University, Manhattan, KS 66502, USA; schaper@vet.k-state.edu
[5] Department of Biomedical Sciences, Rocky Vista University, Ivins, UT 84734, USA; bbrooks@rvu.edu
[6] Department of Research and Scholarly Activity, Rocky Vista University, Ivins, UT 84734, USA
* Correspondence: abrooks@rvu.edu

S1.1 HPLC validation of vancomycin free-base: HPLC was carried out using the following method. Briefly, mobile phase contained 0.1% TFA in water and 0.1% TFA in acetonitrile. The flow rate was set at 1 mL/min. Vancomycin salt stock solution and different fractions during the vancomycin free-base preparation (VancFB2) was analyzed to see if there are by-products. The wavelength was 280 nm and Waters Corporation Alliance e2695 with PDA detector HPLC system (Milford, MA, USA) was used with a C18 column (XTERRA RP 18 5mm 4.6 × 250 mm column).
HPLC validation of V-fb showed that no additional by product was created during the V-fb production. The peaks appeared at the same spot for V-fb as it did for V-HCl (Figure S1).

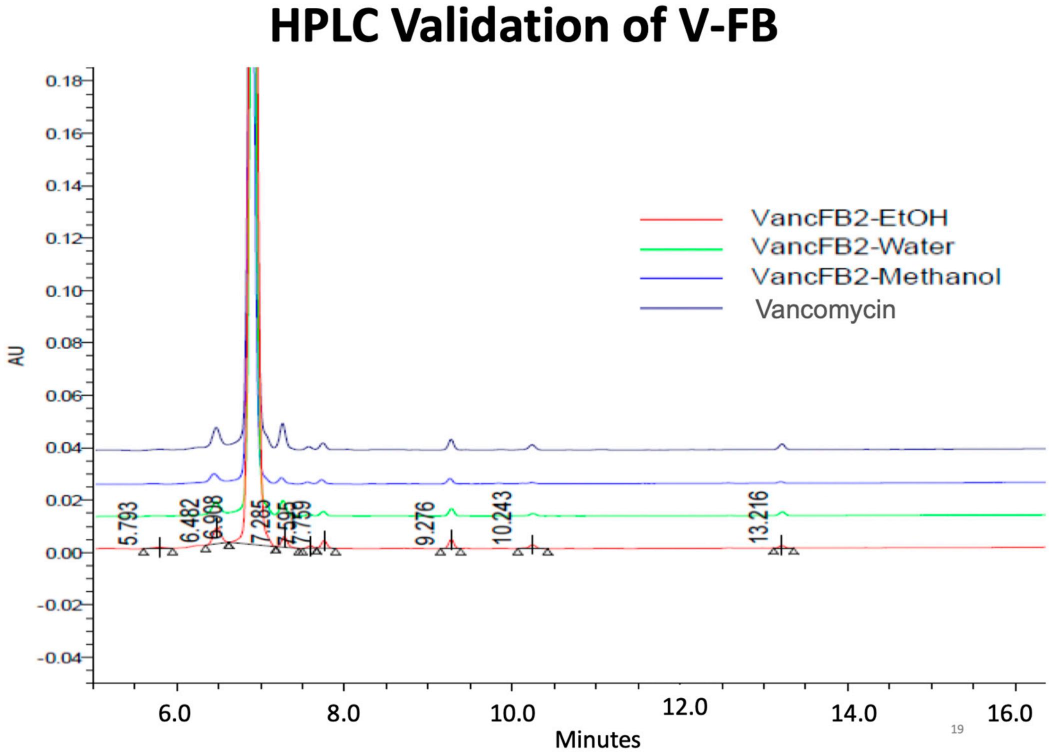


**Figure S1.** HPLC validation of V-fb. The peaks appeared at the same time for both V-HCl and V-fb confirming the production of V-fb.

S1.2 In vitro bioactivity of V-fb: To compare the in vitro bioactivity of prepared V-fb and V-HCl, a Kirby-bauer ZOI (zone of inhibition) assay was done using different concentrations of the drugs against *Staphylococcus aureus* (ATCC 49230) as described in the manuscript. Bioactivity of V-fb and V-HCl against *S. aureus* measured via ZOI assay was similar. No significant difference was seen between the ZOI with no bacterial growth at a particular concentration (n = 3 for each concentration) (Figure S2).

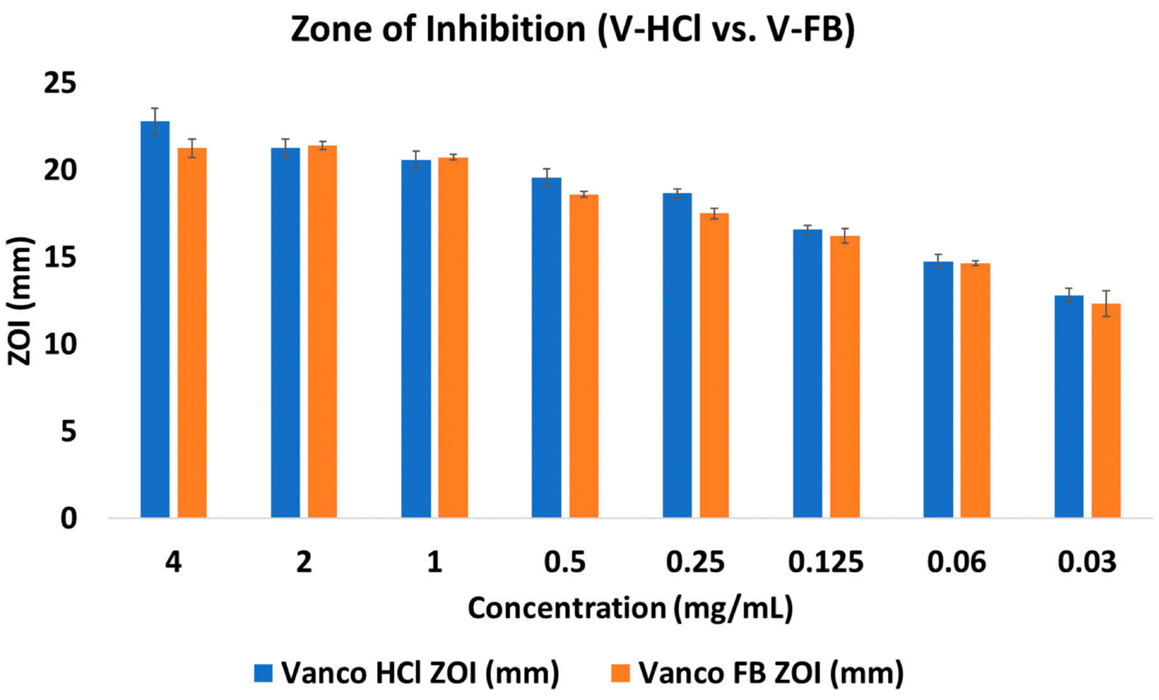


**Figure S2.** The bioactivity of V-HCl and V-FB was determined via a Kirby Bauer zone of inhibition study (Staphylococcus aureus strain 49230: $1.0 \times 10^7$ CFU/mL).

S1.3 Making of Bone Crusher: From a local hardware store three components (Figure S3): the bottom part (A) screws on to the barrel (B). The piston (C) then can be used to pulverize the bone using a hammer. (D) is the finished crusher. The crusher was autoclaved before being used for pulverizing the snap-frozen bone.

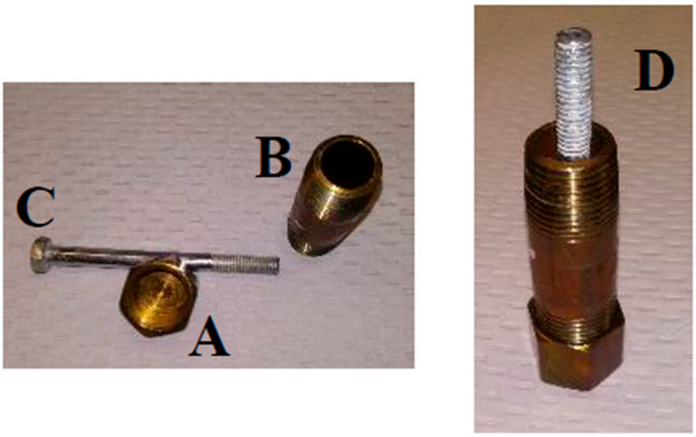


**Figure S3.** The bone crusher was made using three components. The bottom part (**A**) screws on to the barrel (**B**). The piston (**C**) then can be used to pulverize the bone using a hammer. (**D**) is the finished crusher.

**Table S1.** Vancomycin release kinetics from ABVF fitted into different kinetics equation. Korsemeyer-Peppas equation seems to have the best fitted model with high R2 value.

| Drug Release Kinetic Equation | Zero-Order | First-Order | Korsmeyer-Peppas | Higuchi | Hixon-Crowell |
|---|---|---|---|---|---|
| $R^2$ | 0.7181 | 0.9478 | 0.9964 | 0.8939 | 0.907 |
